# Potential association of gender with mortality and withdrawal of life-sustaining therapies in patients with severe TBI: a Canadian multicentre cohort study

**DOI:** 10.1186/cc11107

**Published:** 2012-03-20

**Authors:** AF Turgeon, F Lauzier, A Boutin, N Côte, R Zarychanski, R Fowler, D Scales, M Meade, K Burns, F Bernard, D Zygun, L Moore, D Fergusson

**Affiliations:** 1 Laval University, Quebec, Canada; 2 University of Manitoba, Winnipeg, Canada; 3 University of Toronto, Canada; 4 McMaster University, Hamilton, Canada; 5 Université de Montréal, Canada; 6 University of Calgary, Canada; 7 Ottawa Hospital Research Institute, Ottawa, Canada

## Introduction

Differences in admission patterns, delivery of care and outcomes between women and men admitted to the ICU have been previously identified [1]. However, these observations have not been well described in patients with traumatic brain injury (TBI). Our objective was to identify differences in outcomes between women and men with severe TBI.

## Methods

We used data from a large retrospectively cohort study in which adults with severe TBI (GCS ≤8) admitted to six Canadian level I trauma centres (2005 to 2006) were identified through health records using ICD-10 codes [2]. Demographic, severity of illness, and outcome data were collected by trained abstractors. The primary outcome was the difference in mortality and withdrawal of life-sustaining therapies (WLST) between women and men; secondary outcome was the impact of age (<55 vs. ≥55 years old) among genders. Analyses included chi-square tests and Cox regression analyses adjusted for GCS motor and pupillary reactivity, with stratification for age.

## Results

Among 720 patients, 165 were women (22.9%), 506 (70.3%) aged <55 years old and 214 (29.7%) ≥55 years old. Overall mortality was 31.7% and 70.2% of deaths occurring following the WLST [2]. Unadjusted mortality was 41.2% in women versus 28.8% in men (*P *= 0.003). We observed similar findings in patients <55 years old (30.5 vs. 21.4%, *P *= 0.06), but not among men and women aged ≥55 years old (55.7 vs. 55.0%, *P *= 0.43). Adjusted hazard ratios (HRs) showed a nonsignificantly increased risk of death in women aged <55 years old as compared to men (1.51 (0.92 to 2.47)), and in women aged ≥55 years old (1.53 (0.94 to 2.50)). We observed no difference both in the overall unadjusted incidence of WLST between women and men (73.5 vs. 68.8%, *P *= 0.47) and in women and men aged <55 years old, while there was a nonsignificantly increased rate of WLST in women ≥55 years old (HR 1.53 (0.94 to 2.50)).

## Conclusion

There may be gender-based differences in outcome among patients with severe TBI. Overall, mortality for women tended to be higher, as were decisions for WLST. These differences may be due to unmeasured confounders, biologic responses to TBI, or differences in level of care decision-making.
